# Capabilities and limitations of Pb, Sr and Fe isotopic analysis of iron-rich slags: a case study on the medieval port at Hoeke (Belgium)†

**DOI:** 10.1039/d4ra02887b

**Published:** 2024-07-10

**Authors:** Paulina Biernacka, Marta Costas-Rodríguez, Wim De Clercq, Stijn Dewaele, Johan De Grave, Frank Vanhaecke

**Affiliations:** a Ghent University, Department of Chemistry, Atomic & Mass Spectrometry – A&MS Research Unit Campus Sterre, Krijgslaan 281 – S12 9000 Ghent Belgium frank.vanhaecke@ugent.be; b Historical Archaeology Research Group, Department of Archaeology, Ghent University St.-Pietersnieuwstraat 35 9000 Ghent Belgium; c Centro de Investigación Mariña, Universidade de Vigo, Departamento de Química Analítica y Alimentaria, Grupo QA2 36310 Vigo Spain; d Laboratory for Mineralogy and Petrology, Department of Geology, Ghent University Krijgslaan 281-S8 9000 Ghent Belgium

## Abstract

In this work, an analytical approach was developed for Pb, Sr, and Fe isotopic analysis of archaeological samples recovered from an iron work site by using multi-collector inductively coupled plasma – mass spectrometry (MC-ICP-MS). The sample types include slag, coal, clay and hammer scales, all obtained from an archaeological site at Hoeke (Belgium). Despite the wide concentration range of the target elements present in the samples and some sample manipulations necessarily performed outside of a clean laboratory facility, the analytical procedure yielded accurate and precise results for QA/QC standards while blank levels were negligible. Preliminary results concerning Pb, Sr and Fe isotope ratio variations in archaeological materials associated with iron working processes are provided. The samples revealed high variability in metal isotopic compositions, with the ^208^Pb/^207^Pb ratio ranging from 2.4261 to 2.4824, the ^87^Sr/^86^Sr ratio from 0.7100 to 0.7220, and *δ*^56^Fe values from −0.34 to +0.08‰, which was tentatively attributed to the mixing of materials during the iron production process or variability within the source material. Also, contamination introduced by coal and furnace/hearth lining material could have contributed to the wide range of isotopic compositions observed. Because of the absence of information and data for primary ore samples to compare with, the provenance of the materials could not be established. The present study highlights the challenges in interpreting archaeological data, particularly in terms of the isotopic variability observed. It underscores the necessity of integrating analysis data with historical and archaeological knowledge. Further research, involving detailed analysis of these source materials combined with robust historical evidence, is essential to validate hypotheses concerning the origin of iron.

## Abstract 2

An analytical approach was developed for Pb, Sr, and Fe isotopic analysis of archaeological samples recovered from an iron work site by using multi-collector inductively coupled plasma – mass spectrometry (MC-ICP-MS).

## Graphical abstract

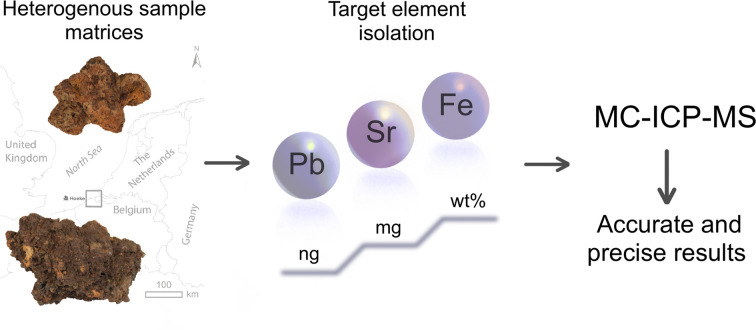

## 1. Introduction

One of the fundamental queries in archaeology is establishing the origin of the raw materials used in the making of various types of artefacts. By determining the provenance of such raw materials (*e.g.*, ores), patterns of trade and exchange can be revealed.^1^ The knowledge of the origin of metal artifacts and the raw materials they were manufactured from helps unravel the relationship between primary and secondary ironmaking sites and provides insight into specialized trade routes. The observation of transfer of such materials between locations provides evidence of human interaction and the exchange of goods, services, and ideas, offering insights into past social relations, economic structures, and mobility patterns.^2^

However, analysis of archaeological metal artefacts can be challenging due to the nature of the material itself (complex and heterogeneous samples) and potential degradation of the material over time. The situation becomes even more complex when so-called secondary metals are mixed within a system. For example, the metal used for manufacturing an artifact could have been obtained by remelting of other damaged objects that were made from metals originating from different ores. Additionally, technological processes such as smelting or roasting can lead to the loss of specific elements (Sb, Zn, As) altering the overall elemental composition, while isotope fractionation accompanying some of these processes could also affect the isotopic composition of some constituting elements. The archaeological samples could have undergone different physicochemical processes (*e.g.*, corrosion, post-depositional processes), rendering obtaining reliable information difficult.^3,4^ Nevertheless, some studies showed negligible isotope fractionation (within experimental error) for metal objects made of, for instance, Fe, Sn or Pb.^5–7^

Different approaches based on the use of (trace) element patterns have been widely used for assessing the provenance of metal artefacts.^8,9^ However, interpretation of such elemental fingerprints is often not straightforward, especially when dealing with elements that have different affinities for metal and slag. Elements such as Co and Ni, known as siderophile elements, are absorbed by the metal, while lithophile elements (*e.g.*, Ca and Sr) tend to enter in the slag.^10^ Therefore, a direct comparison between the composition of a metal artefact with that of the ore source may be very difficult, especially in the case of iron.^11^ Also elemental signatures of slag inclusions can be involved in the analysis, but these inclusions can also become altered by contamination and may originate from various sources, such as the surrounding soil, coal ashes, and local building materials.^8,12^ Chemical analysis of slag inclusions using laser ablation – inductively coupled plasma-mass spectrometry (LA-ICP-MS) has led to a major progress in this context, but limitations associated to elemental fractionation, matrix effects and spectral interferences are still encountered.^13,14^

Stable isotopic analysis of metals in different specimens, such as objects, by-products, and ores, *via* multi-collector (MC) ICP-MS is increasingly used for tracing the geographical origin of artefacts.^15–17^ Instrumental advances in MC-ICP-MS allow to address challenging archaeological applications due to improvements in sensitivity, enhanced sample throughput, and simplified sample preparation procedures (especially when using laser ablation).^18^ By examining the ratios of stable isotopes of (a) selected target element(s) present in the material, valuable information on the source from which the metals were derived can sometimes be obtained as trace elements are typically not sufficient to resolve a provenance issue. However, different parts of the same sample, as well as ore bodies, may show variation in the isotopic composition of such target element, complicating the task of obtaining representative data that accurately reflect the overall isotopic fingerprint of the specimen.^19,20^ In addition, some metals can be present at low concentration in the samples, making precise isotope ratio measurements challenging.^21^ The combination of elemental, isotopic and spatially resolved analysis can be a valuable tool for addressing current challenges in archaeological provenancing.

Lead isotope ratios have already been used for providing insight into the provenance (geographical origin) of metal ores used as raw materials, particularly for ancient bronze objects, but its use for iron slag samples is still debated.^22^ The advantage of Pb isotopic analysis in an archaeometric context, is that Pb isotopes do not fractionate during high-temperature processes such as roasting or smelting, as a result of which Pb isotope ratios do not undergo significant changes. This allows to trace the provenance of the ore more reliably.^14,23,24^ However, when the Pb concentration is low and the matrix contains high levels of other metals, re-evaluation of the sample preparation and isotope ratio measurement protocols is advisable.^25^

Strontium isotopic analysis is also a potential tool for tracing the origin of both contemporary and archaeological materials, ranging from ceramics and glass to remains of living species, including humans. While the former applications (ceramics and glass) rely on comparison of the ^87^Sr/^86^Sr isotope ratio of the objects with that of raw materials of various origin potentially used for their production, the latter is based on the fact that Sr from the geological bedrock gradually moves into soil and groundwater, eventually entering the biosphere and food chain.^18,26,27^ However, in the context of the present study, it has to be taken into account that the Sr isotopic composition of a metal artefact can be affected by that of the materials used for building the furnace used for metal production, especially the clay used for the furnace lining.^11^

Osmium isotopic analysis has also been suggested as a promising tool for metal provenancing studies.^28,29^ However, a substantial amount of sampled material is required for its isotopic analysis, and there is a risk of Os loss due to the strong oxidation conditions during sample digestion, which can affect the reliability of the results.^30^

The use of iron isotope ratios for determining the provenance of metal has not been extensively investigated in the past. However, recent studies have begun to explore the application of iron isotopic analysis as a tool for studying the origin of ancient iron objects. These studies have highlighted several advantages of this approach, including the absence of iron isotope fractionation during iron production operations and the requirement of only a small sample size for analysis. However, also some limitations were indicated, such as the natural variability of the iron isotope ratios in some ore bodies, such as those found in bog iron ore deposits.^31,32^

Provenance studies predominantly rely on the use of a single isotopic system. However, significant overlap between the signatures for raw materials stemming from various possible locations of origin often occurs.^33^ Therefore, the use of multiple isotopic systems can provide a more precise answer as to the provenance of the samples under study.^34^ However, analytical development is often needed for such purpose because: (i) the target elements may be present in a wide range of concentrations or at (ultra)trace level only; (ii) the sample matrices are often complex, potentially leading to spectral interferences, while they often display a large degree of heterogeneity. This work aimed to evaluate the use of three isotopic systems, *i.e.* those of Pb, Sr and Fe, for the provenancing of ancient iron-rich slags, with this manuscript placing particular emphasis on methodological aspects.

**Fig. 1:**
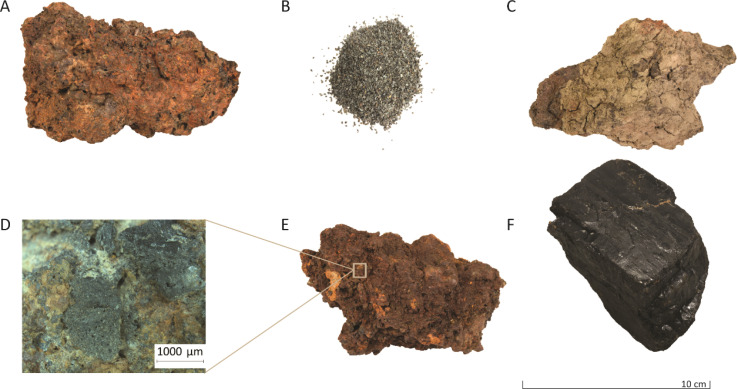
Representative photographs of the investigated material found at the Hoeke site: (A) iron slag, (B) hammer scales retrieved from the soil, (C) iron slag sample with clay attached to the surface, (D) close-up view (1000 μm) of a coal fragment adhered to the surface of an iron slag sample (E), and (F) a coal specimen.

The iron-rich slags investigated in this work were obtained from an archaeological site at Hoeke, Belgium. Hoeke was one of the outer harbours of Bruges, located along the Zwin tidal inlet, which, during medieval times, linked the city to other medieval cities in Europe. During a geophysical survey on a 12 hectare area of the former harbour region of Hoeke, conducted using an Electromagnetic Induction (EMI) sensor, strong signals suggested the presence of remnants of iron-working activities.^35^ Excavations in 2018 and 2021 confirmed these expectations, and large quantities of iron slags, hammer scales, charged materials (charcoal, coal), and other waste products were found (Fig. 1). Since Hoeke was a harbour town, ships coming and leaving were maintained and repaired at the site, which explains the former occurrence of iron-working activities.

The large number of waste products stemming from the iron production provides a unique opportunity to investigate the local iron production process, which has not been previously addressed. Geochemical analysis of these samples revealed various technological aspects of iron production, including the occurrence of smithy activity.^36^ As there is no on-site evidence of ore extraction, the primary hypothesis is that the iron discovered on site was sourced from (an) external region(s). Historical sources indicate that during the high medieval period, goods such as metals, timber and foods were commonly imported from Hanseatic cities, as records indicate that these areas were known for their metal production and trade.^37,38^

## 2. Materials and methods

### 2.1. Samples

The sample set consisted of iron slags, clay fragments attached to the surface of slags, hammer scales and coal fragments. Fig. 1 illustrates some of the samples.

The mineralogical composition of the samples (except for coal) was determined using a Philips PW3710 X-ray diffractometer (current 30 mA, voltage 40 kV), equipped with a cobalt anode X-ray tube and scanned at a 2*θ* angle from 3° to 70°. The step size was set at 0.020° with a residence time per step of 2.5 s. Additionally, the mineralogy of the samples was also studied by using reflected light microscopy (Nikon Eclipse Ni-E motorised microscope equipped with a Nikon DS-Ri2 camera). Iron slags from Hoeke mainly consist of quartz, a mixture of iron oxides, such as magnetite Fe_3_O_4_ and wüstite FeO, as well as fayalite FeSiO_4_, and iron aluminium oxide. Clay samples are mainly composed of quartz and silicate minerals such as laihunite Fe^2+^Fe_2_^3+^(SiO_4_)_2_ and anorthoclase (Na, K)AlSi_3_O_8_. Hammer scales are solely composed of quartz and magnetite.^36^

### 2.2. Reagents

All solutions were prepared with ultrapure water (resistivity ≥ 18.2 MΩ cm), produced using a Milli-Q Element water purification system (Millipore, France). Trace metal analysis grade PrimarPlus 14 M nitric acid (HNO_3_) and 12 M hydrochloric acid (HCl) acquired from Fisher Chemicals (UK) were further purified *via* sub-boiling distillation in a Savillex® DST-4000 acid purification system (Savillex Corporation, United States). Hydrofluoric acid (HF, 48%) was purchased from Merck ( Germany) and used as such.

Single-element standard solutions (1000 mg L^−1^) of Ca, Fe, Pb, Sr and Ga used for quantification purposes were acquired from Chem-Lab NV (Belgium) and those of Al and Ti from Inorganic Ventures (the Netherlands).

NIST SRM 987 SrCO_3_ isotopic reference material was obtained from the National Institute for Standards and Technology (NIST, USA) and used in the Sr MC-ICP-MS isotopic analysis.

NIST SRM 981 isotopic reference material was used in the Pb MC-ICP-MS isotopic analysis. NIST SRM 997 Tl isotopic reference material was used as internal standard for correction of instrumental mass discrimination. A previously characterized standard solution of Pb (Inorganic ventures, lot G2-PB03044) was used as an in-house standard for quality assurance and quality control (QA/QC) of the Pb isotope ratio measurements.

IRMM-524A isotopic reference material (Institute for Reference Materials and Measurements–IRMM, Belgium) was used in the Fe MC-ICP-MS isotopic analysis. A solution of Ni (Inorganic Ventures) was used as internal standard for correction of instrumental mass discrimination. A standard solution of Fe (Inorganic ventures, lot D2-FE03110) was used as in-house isotopic standard for QA/QC purposes.

The resin used for the isolation of Sr and Pb from the sample matrices was Sr-Spec (Sr_B50-A 100–150 μm from Triskem International, France), while for the isolation of Fe, AG MP-1 anion exchange resin (100–200 μm purchased from Bio-Rad, USA) was used. The resins were stored in polyethylene tubes filled with Milli-Q water prior to use.

### 2.3. Cleaning protocols and sample manipulation

Major elements, such as Fe, Si, Ca, and K, are present at weight percentage (wt%) levels in iron slags (Table 1). As a result, sample preparation could not be carried out in the UGent-A&MS clean lab due to the high risk of contamination and interference with (especially biomedical) applications involving trace amounts of especially Ca, Fe and K.

**Table:** Average elemental oxide composition (determined *via* portable X-ray fluorescence spectrometry – pXRF) of the examined slags selected. Results expressed in wt%. LE = light elements

	LE	Al_2_O_3_	CaO	CuO	FeO	K_2_O	MnO	P_2_O_5_	SO_3_	SiO_2_	SrO	TiO_2_	ZrO_2_
wt%	48.58	2.55	1.06	0.01	22.34	1.40	0.05	0.08	0.22	6.24	0.01	0.11	0.01
SD	0.23	0.95	0.67	0.02	9.76	0.36	0.10	0.03	0.21	3.96	0.01	0.06	0.01

As a consequence, the samples had to be processed in a common laboratory. A problem arose with one of the target elements (Pb), as its concentration in the samples was very low compared to the concentrations of Sr and, especially, Fe. Following the isolation procedure, a significant contribution of the procedure blank to the Pb concentration was observed, making it impossible to obtain accurate isotope ratio data. As a compromise between the use of a clean laboratory and a common laboratory, an evaporation box (Quimipol, Spain) especially designed for low-level work, manufactured from PMMA and equipped with a PP fan rotating at 3000 rpm and a H14 HEPA filter, located in a common laboratory was installed to minimise contamination. The aim was to mimic the conditions of a clean laboratory to the largest possible extent while working in a common laboratory setting. The entire procedure, including digestion, evaporation to dryness, target element isolation, and sample dilution, was performed within this specially designed evaporation box. Under these conditions, the Pb blank level decreased significantly. The Pb blank level after the first chromatographic separation performed under the fume hood in the common laboratory was *ca.* 0.7 μg, while following the same procedure but inside the evaporation box, the Pb blank level was reduced by more than two orders of magnitude to 0.004 μg.

Large variation in sample composition and the wide range of the target element concentrations in the objects of study, *i.e*. from a few ng of Pb to wt% of Fe, also necessitated the use of proper cleaning protocols to avoid potential (cross-)contamination. PFA screwcap beakers (Savillex Corp., USA) used for the digestion procedure were pre-cleaned using the 7-step cleaning procedure outlined in Table 2. Polypropylene (PP) material was soaked two times for 24 h, first in 1.2 M HCl and subsequently in Milli-Q water at 110 °C. Final dilutions and cleaning of labware were performed in a metal-free class-10 clean lab facility (Picotrace, Germany) at UGent-A&MS.

**Table:** Cleaning protocol for PFA beakers

Step	Reagent	Duration	Temperature
1	Reverse *aqua regia*	24 h	110 °C
2	Soap solution (NovaClean™)	24 h	110 °C
3	HNO_3_ (7 M, trace analysis grade)	24 h	110 °C
4	HNO_3_ (7 M, trace analysis grade)	24 h	110 °C
5	HCl (6 M, trace analysis grade)	24 h	110 °C
6	HCl (6 M, trace analysis grade)	24 h	110 °C
7	HCl (1.2 M, UP)	24 h	110 °C

### 2.4. Sample pre-treatment

**Fig. 2:**
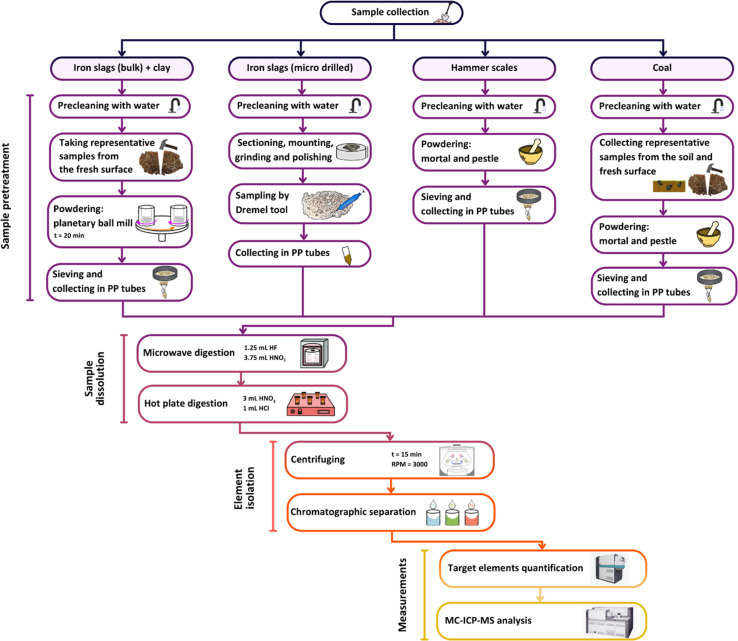
Flowchart of the analytical protocol.

Iron slag samples were collected for analysis using two sampling approaches: bulk sampling and micro-drilling. A scheme of the procedure is presented in Fig. 2. Potential contamination during sample preparation could arise from the sampling of heterogeneous slag pieces, such as slag that has been physically mixed with coal or clay material, or could originate from the soil. Thus, for bulk analysis, the selected slag material was first pre-cleaned with water and subsequently, sub-samples were manually broken off, thus enabling representative pieces of slag to be extracted from a fresh surface, visually not exhibiting any traces of weathering and/or post-depositional processes. Then, the slags were crushed using a hammer (contamination was avoided during this phase by wrapping the sample in plastic) and subsequently grinded to a fine powder using a Retsch planetary ball mill (at the Department of Geology of Ghent University) for around 20 minutes. Finally, the powder obtained was sieved at 100 μm (Retsch sieve) and collected in metal-free PP tubes. To minimize the risk of contamination during each step, all equipment was thoroughly precleaned with Milli-Q water, and the ball mill was additionally cleaned by processing quartz.

A second approach of sampling consisted of micro-drilling at polished sections of the iron slags using a Dremel 4000 tool equipped with a diamond step drill bit. To avoid mixtures of different materials (such as coal and clay fragments), homogenous parts of slag were selected only. After each sampling, the drill bit was cleaned with a solution of 3% HNO_3_, followed by rinsing with Milli-Q water.

Hammer scales were retrieved from the soil samples, rinsed with Milli-Q water and then grinded to powder in an agate mortar.

Clay samples were subjected to the same sample pre-treatment as used for bulk analysis of iron slags.

Coal pieces were extracted both from the fresh surface of iron slags and taken up from the soil as individual pieces, which were subsequently crushed and powdered in an agate mortar.

#### 2.4.1. Sample dissolution

All samples were acid-digested using a high-pressure Multiwave 7000 microwave unit (Anton Paar, Austria), equipped with a PTFE-TFM rack for eighteen 10 mL pressure-sealed vials (PTFE-TFM, HF-resistant) in a stainless steel microwave digestion cavity with a PTFE-TFM liner, pressurised with N_2_. The maximum operating parameters that the system can operate at are 300 °C, 199 bar and 1700 W. About 500 mg of sample powder was weighed in a microwave (MW) vial, and a mixture of concentrated HF and HNO_3_ (1.25 mL and 3.75 mL, respectively) was added. The samples were processed in a batch of 18. Then, the vessels were placed inside the liner filled with the loading solution (0.42 M HNO_3_) and the microwave cavity was pressurised at 40 bar for digestion. The microwave program consisted of the following steps: (1) 12 min ramp to 240 °C at 140 bar followed by (2) 30 min at 240 °C at 140 bar. After cooling down, the digests obtained were transferred into PFA Savillex beakers and dried at 90 °C. Afterwards, the samples were re-dissolved and subjected to a second acid digestion on a hotplate using 3 mL of concentrated HNO_3_ and 1 mL of concentrated HCl for 24 h at 110 °C. The samples were again evaporated to near-dryness and dissolved in 2 mL 7 M HNO_3_ for the subsequent chromatographic separation. For some samples, a residue was present in the beaker after the two-step digestion, which most likely consisted of fly ash and/or refractory minerals. After redissolving the residue obtained upon evaporation, the solution was centrifuged for 15 min at 3000 rpm, the supernatant collected and immediately loaded onto the chromatographic column.

#### 2.4.2. Chromatographic target element isolation

The chromatographic separation protocols are shown in Table 3. The resin was first dispersed in Milli-Q water, then washed a few times with 7 M HNO_3_ (UP) and 6 M HCl (UP), and finally with Milli-Q water. Then, a pre-cleaned column was filled with 400 μL of Sr-Spec resin and sequentially washed with 20 mL of Milli-Q water, 4 mL of 7 M HNO_3_, 1 mL of 6 M HCL and finally 20 mL of Milli-Q water. The column was subsequently conditioned with 2 mL of 7 M HNO_3_. Afterwards, 1.8 mL of sample dissolved in 7 M HNO_3_ was loaded onto the column. Matrix elements were removed using 5 mL of 7 M HNO_3_, after which the Sr fraction was eluted using 6 mL of 0.05 M HNO_3_ and collected in a PP tube. Subsequently, the column was conditioned with 1 mL of 3 M HCl, after which Pb was eluted using 6 mL of 8 M HCl and collected in a Teflon Savillex® beaker. The Pb fraction was evaporated to dryness and redissolved in 1 mL of 7 M HNO_3_. Then, the Pb fraction was submitted to a second chromatographic separation, carried out using the same column and the same procedure. The pure Pb fraction thus obtained was evaporated to dryness and redissolved in 500 μL of 0.28 M HNO_3_.

**Table:** Elution sequence for chromatographic isolation of Sr, Pb and Fe

Step↓	Sr and Pb		Fe	
	Eluent	Volume [mL]	Eluent	Volume [mL]
Washing	Milli-Q	20	7 M HNO_3_	10
	7 M HNO_3_	4	Milli-Q	10
	6 M HCl	1	0.7 M HNO_3_	10
	Milli-Q	20	Milli-Q	10
Conditioning	7 M HNO_3_	2	8 M HCl + 0.1 mM H_2_O_2_	5
Sample loading	7 M HNO_3_	1.8	8 M HCl + 0.1 mM H_2_O_2_	5
Matrix removal	7 M HNO_3_	5	8 M HCl + 0.1 mM H_2_O_2_	3
			5 M HCl + 0.1 mM H_2_O_2_	12
Target element elution	0.05 M HNO_3_ (Sr collection)	6	0.7 M HCl	10
	3 M HCl (change of medium)	1		
	8 M HCl (Pb collection)	6		

The potential presence of matrix elements such as Al, Mg, Ca and Fe in the purified Sr and Pb fractions was monitored by single-collector ICP-MS to ensure sufficient purity. After the first Pb isolation, some of these elements still remain in the Pb fraction such that a two-step isolation protocol was required.

For Fe isolation, an aliquot of the sample digest was first diluted (10^7^-fold) to avoid saturation of the resin. The chromatographic separation was carried out using 2 mL of AG-MP-1 anion exchange resin which was precleaned with 10 mL of 7 M HNO_3_, 10 mL of Milli-Q water, 10 mL of 0.7 M HNO_3_ and 10 mL of Milli-Q water and conditioned with 5 mL 8 M HCl + 0.1 mM H_2_O_2_. The sample was loaded onto the column and the matrix was eluted using 3 mL of 8 M HCl + 0.1 mM H_2_O_2_ followed by 12 mL of 5 M HCl + 0.1 mM H_2_O_2._ Afterwards, Fe was eluted using 10 mL of 0.7 M HCl and collected in a Teflon Savillex® beaker. The Fe fraction was evaporated to dryness at 90 °C and redissolved in 500 μL of 0.28 M HNO_3_.

### 2.5 Instrumentation and measurements

Pb, Sr and Fe isotope ratio measurements were accomplished using a Neptune *Plus* MC-ICP-MS instrument (ThermoScientific, Germany), equipped with a high-transmission Jet interface (Jet-type Ni sampling cone and X-type Ni skimmer cone, 1.1 mm and 0.8 mm *∅* aperture, respectively). A conventional sample introduction system, composed of a 100 μL min^−1^ concentric nebulizer mounted onto a dual spray chamber with a cyclonic and a Scott-type sub-unit, was used for Sr and Fe isotope ratio measurements. The Aridus II desolvator system (Teledyne CETAC Technologies Inc., USA), equipped with a 100 μL min^−1^ PFA C-type nebulizer was used for Pb isotope ratio measurements. The instrument settings and data acquisition parameters are compiled in Table 4.

Instrument settings and acquisition parameters for the Neptune MC-ICP-MS instrument

**Table:** 

Instrument settings		Sr isotopic analysis	Pb isotopic analysis	Fe isotopic analysis
		Wet plasma	Dry plasma^a^	Wet plasma
RF power, W		1200	1200	1200
Gas flow rates, L min^−1^	Sample	1.050–1.090^b^	1.030–1.050^b^	1.050–1.070^b^
	Auxiliary	0.70–0.90^b^	0.70–0.90^b^	0.70–0.90^b^
	Cool	15	15	15
	Sweep	—	7.5	—
	N_2_	—	0.002	—
Resolution mode		Low^c^	Low^c^	Medium^c^
Typical sensitivity		20 V for ^88^Sr at 100 μg L^−1^ Sr	1 V for ^208^Pb at 10 μg L^−1^ Pb	15 V for ^56^Fe at 300 μg L^−1^ Fe

a Dry plasma conditions obtained using the ARIDUS II sample introduction system. The temperatures of the spray chamber and membrane desolvator were 110 and 160 °C, respectively.

b Optimised daily for maximum intensity.

c Pseudo-high mass resolution: in the equation for mass resolving power *m*/Δ*m*, Δ*m* is defined as the difference between masses corresponding to 5 and 95% of the signal intensity at the plateau. A resolving power of 3800 was measured for the medium mass resolution mode.

**Table:** 

Data acquisition parameters			
Mode	Static, multi-collection	Static, multi-collection	Static, multi-collection
Idle time, s	3	3	3
Integration time, s	4.194	4.194	4.194
Number of integrations	1	1	1
Number of blocks	1	1	1
Number of cycles per block	30	60	45
Baseline	300 s baseline every 20 samples	300 s baseline every 20 samples	300 s baseline every 20 samples

**Table:** 

Cup configurations							
Sr cup configuration	L4	L3	L2	L1	C	H1	H2
Nuclide	^82^Kr	^83^Kr	^84^Sr	^85^Rb	^86^Sr	^87^Sr	^88^Sr
Amplifier	10^11^ Ω	10^11^ Ω	10^11^ Ω	10^11^ Ω	10^11^ Ω	10^11^ Ω	10^11^ Ω
Pb cup configuration	L3	L2	L1	C	H1	H2	H3
Nuclide	^202^Hg	^203^Tl	^204^Pb	^205^Tl	^206^Pb	^207^Pb	^208^Pb
Amplifier	10^11^ Ω	10^11^ Ω	10^13^ Ω	10^11^ Ω	10^13^ Ω	10^13^ Ω	10^13^ Ω
Fe cup configuration	L4	L2	L1	C	H1	H3	
Amplifier	10^11^ Ω	10^11^ Ω	10^11^ Ω	10^11^ Ω	10^11^ Ω	10^11^ Ω	
Nuclide	^54^Fe	^56^Fe	^57^Fe	^58^Fe, ^58^Ni	^60^Ni	^62^Ni	

An acid blank (0.28 M HNO_3_) and procedural blanks treated in the same way as the samples were measured at the beginning of each measurement sequence to evaluate their contribution to the signal intensities. Three procedural blanks were always included in each batch of samples. Isotope ratio measurements for Pb, Sr and Fe were performed at 10 μg L^−1^, 100 μg L^−1^ and 300 μg L^−1^ concentration levels, respectively.

Prior to MC-ICP-MS measurements, quantification of the target elements was performed using a Thermo Scientific Element XR (Germany) single-collector sector-field ICP-MS unit, relying on external calibration, with Ga and Tl as internal standards to correct for potential matrix effects and/or instrument instability. Sample introduction was accomplished using a 200 μL min^−1^ quartz concentric nebulizer mounted onto a cyclonic spray chamber.

For the ^87^Sr/^86^Sr ratio, the correction for instrumental mass discrimination was accomplished using internal correction following Russell's law using an ^88^Sr/^86^Sr ratio of 8.375209 ^39^ and subsequent external correction using isotopic reference material (NIST SRM 987) measured in a sample-standard bracketing (SSB) approach.^40^ The intensities for ^83^Kr^+^ and ^85^Rb^+^ were monitored and used to correct for the contributions of Kr at *m*/*z* = 84 and 86, and Rb at *m*/*z* = 87 respectively.

For the Pb isotope ratios, the instrumental mass discrimination was corrected for using the method described by Baxter *et al.*, using spiked Tl as an internal standard. In addition, external correction was applied as well using NIST SRM 981 measured in a SSB approach.^41^ The signal of ^204^Pb was corrected for interference from ^204^Hg based on the signal intensity for ^202^Hg.

For the Fe isotope ratios, instrumental mass discrimination was corrected for using the method described by Baxter *et al.*, using Ni as internal standard and external correction based on IRMM-524A measured in a SSB approach.^41^

Data statistical analysis was performed using the Software Package for Statistical Analysis (SPSS) version 29 and Microsoft Excel (Version 2404).

## 3. Results

### 3.1. Method evaluation

The samples from this study are characterized by a large heterogeneity in composition, with Fe concentrations ranging from 0.90 to 72 wt% while the Pb concentration varied from less than 1 μg g^−1^ to about 200 μg g^−1^ and the Sr concentration from 0.008 to 222.9 mg g^−1^.

As the Pb concentration was very low compared to those of other matrix/target elements, the use of a two-step isolation procedure was required for the efficient removal of matrix elements. After two column passages, the contributions of Al, Sr, Mg, Ca and Fe in the pure Pb fraction were less than 1% of the Pb content in all cases.

To the best of the authors' knowledge, there is no reference material available for this sample type and therefore a geological certified reference material, G-3 granite (United States Geological Survey, USGS), was used instead for method evaluation. The values obtained were 18.387 ± 0.0074 for the ^206^Pb/^204^Pb ratio, 0.8497 ± 0.0001 for the ^207^Pb/^206^Pb ratio and 2.1143 ± 0.0003 for the ^208^Pb/^206^Pb ratio, in good agreement with previously reported data (^206^Pb/^204^Pb = 18.390 ± 0.079; ^207^Pb/^206^Pb = 0.850 ± 0.043; ^208^Pb/^206^Pb = 2.113 ± 0.071).^42^

The procedural blanks, that were also subjected to the sample digestion and chromatographic isolation protocols, were analysed in the same way as the samples. In each batch of samples consisting of ≈18 samples, three different blanks were always measured at the beginning of the experiment. Blank signals were always negligible compared to the Pb, Sr and Fe intensities obtained for the sample solutions analysed (≤1% in all cases).

The in-house isotopic standard solutions were included in each sequence for QA/QC purposes. Results obtained for the Pb in-house standard were 2.1508 ± 0.0001 for the ^208^Pb/^206^Pb ratio and 0.9037 ± 0.0001 for the ^207^Pb/^206^Pb ratio (*n* = 38), in agreement with data reported in previous papers (^208^Pb/^206^Pb = 2.15331 ± 0.00003 and ^207^Pb/^206^Pb = 0.90413 ± 0.00002).^43^ For Fe, the mean *δ*^56^Fe value of the in-house standard was 0.47 ± 0.09‰, which was in good agreement with previously reported data (*δ*^56^Fe = 0.45 ± 0.04‰).^44^

### 3.2 Pb, Sr and Fe isotopic signatures of iron-rich slags

Lead and strontium isotopic signatures of the iron slags, hammer scales, clay samples, and coal are compiled in the ESI† (Table S1) and the Fe isotope ratios in Table S2.† The precision, expressed as 2SD (sample preparation replicates, *N* = 60) was 0.011–0.048 for the ^206^Pb/^204^Pb, ^207^Pb/^204^Pb, and ^208^Pb/^204^Pb ratios and 0.002 for the ^207^Pb/^206^Pb and ^208^Pb/^206^Pb ratios, respectively. The precision obtained for the ^87^Sr/^86^Sr ratio was 0.0001 (2SD) and for the *δ*^56^Fe and *δ*^57^Fe values, the precision was 0.28‰ and 0.42‰, respectively.

**Fig. 3:**
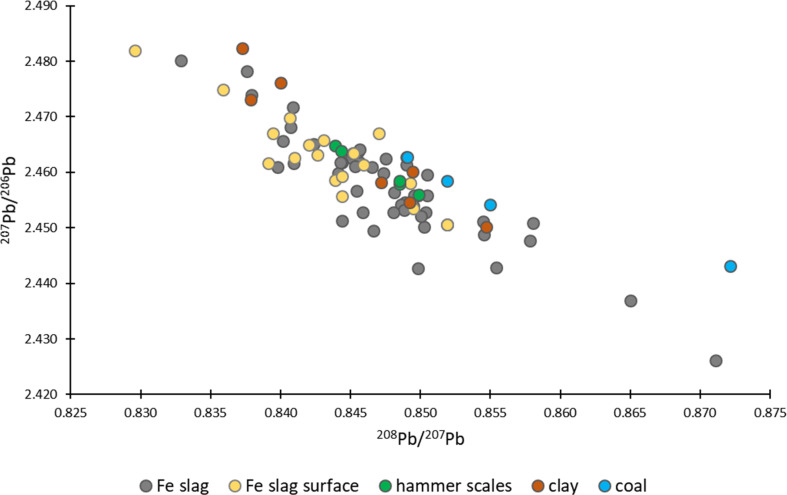
Overview of the Pb isotope ratios obtained for the different types of material investigated: Fe slag, Fe slag surface, hammer scales, clay and coal. The error bars, indicating standard deviations range between 0.0001 and 0.0078, are overlapped by the markers.

Lead isotope ratios exhibit large variations and did not cluster together by sample type (Fig. 3). Additionally, there is a significant overlap of the values obtained for the surface of iron slags and for the corresponding bulk samples (*t*-test, *p* > 0.05), although the bulk slag samples show a larger spread. Hammer scales and clay samples showed Pb isotopic signatures similar to those of the iron slags. Coal samples, on the other hand, showed a slightly heavier Pb isotopic signature compared to the other materials, however, this difference was not significant (*t*-test *p* > 0.05).

**Fig. 4:**
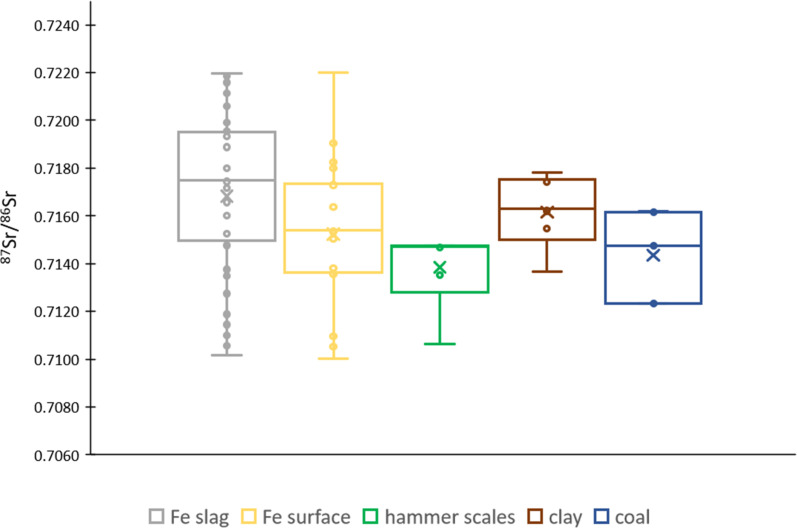
Box plot showing the ^87^Sr/^86^Sr ratio for the different types of material investigated – Fe slag, Fe slag surface, hammer scales, clay and coal. The average SD is 0.0001.

Similarly to the Pb isotope ratios, also the ^87^Sr/^86^Sr isotope ratio showed a marked spread. Data for Sr are presented in Fig. 4 and Table S1.† The Sr concentration ranged between 0.008 and 222.9 mg g^−1^ and the ^87^Sr/^86^Sr ratio between 0.7100 and 0.7220. Iron slags and clay showed a slightly more radiogenic ^87^Sr/^86^Sr isotope ratio compared to that of the surface of iron slags, hammer scales, and coal. However, all results fall within the range obtained for the iron slags, indicating a non-distinctive Sr isotopic signature.

**Fig. 5:**
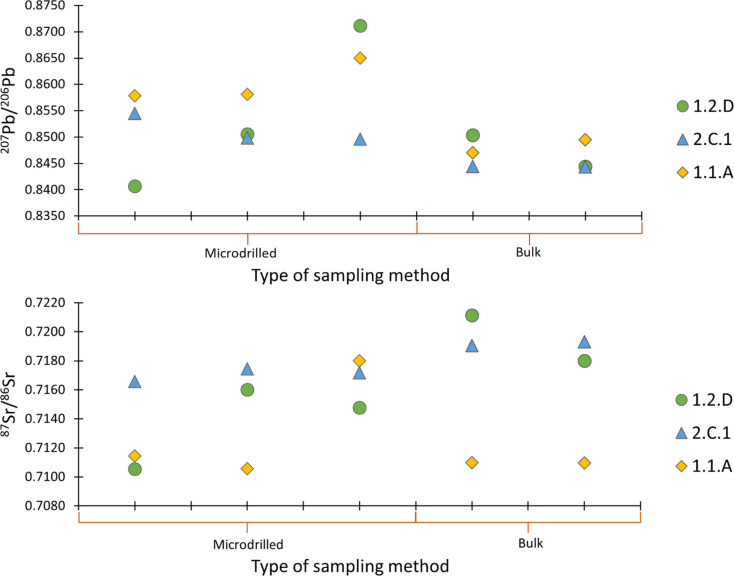
A visual representation of the Sr and Pb isotopic heterogeneity within one sample. In this case, one sample was measured five times, three measurements were performed on micro-drilled material and two measurements were carried out on the bulk sample. The average SD is 0.0001 and 0.0007 for the ^87^Sr/^86^Sr and ^207^Pb/^206^Pb isotope ratios, respectively.

To explore the variability within a sample and assess representativeness of the Pb and Sr isotopic signatures of the bulk material, both bulk and micro-drilled specimens were analysed for selected samples. Fig. 5 illustrates isotopic signatures for sub-samples of the same material. As can be observed, significant variations were established, particularly in samples 1.1.A and 1.2.D. In sample 1.2.D, the ^208^Pb/^207^Pb values range from 0.8406 to 0.8711 reflecting a considerable disparity and the ^87^Sr/^86^Sr ratio from 0.7105 to 0.7211. The precisions (SD) obtained for the Pb isotope ratio of the bulk and micro-drilled samples were 0.0014 and 0.0061, respectively and for Sr 0.0006 and 0.0020.

**Fig. 6:**
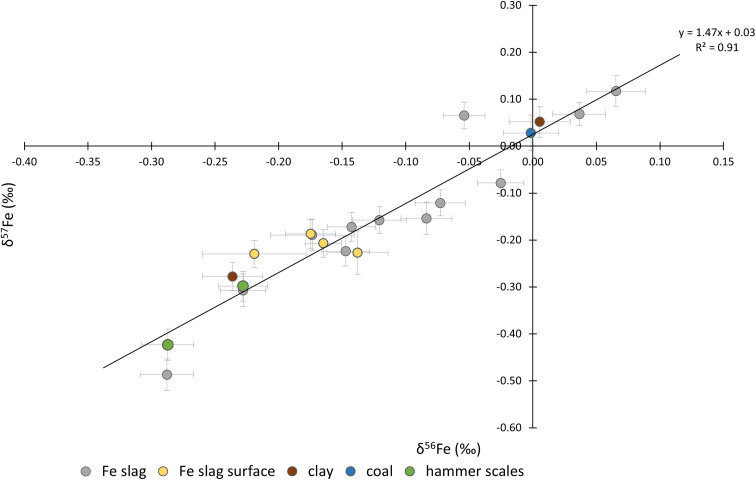
Three-isotope plot for Fe for the different types of material investigated: Fe slag, Fe slag surface, hammer scales, clay and coal. Three samples did not follow the fractionation line and thus were not included in the graph.

The *δ*^56^Fe values ranged between 0.08 and −0.34‰ and the *δ*^57^Fe values between 0.16 and −0.48‰. The Fe three-isotope plot is presented in Fig. 6. As can be seen, the data plot along the theoretical mass fractionation line.

## 4. Discussion

A common approach for tracing the provenance of raw materials used in the manufacturing of objects is the comparison of the isotopic composition of constituting elements to those within potential source materials.^1^ An important prerequisite for such an approach to be useful is that the isotopic composition of the targeted elements has not been affected by isotope fractionation during processing. In the conceptualisation of this work, a comparison of the isotopic composition of selected elements present in iron slags and hammer scales with those in the potentially used ore samples was considered. However, no ore samples were found within the excavation, preventing a direct comparison and assessment of potential fractionation during the ore processing.

However, provenancing not only relies on comparing isotopic data with source material available for analysis, but also relies on the consultation of archaeological and historical records. Considering the late medieval period, the period from which the samples stem, one of the possible scenarios is that during the Hanseatic period in Europe, ore was brought to Flanders from other European locations *via* trade. The Hanseatic League played a significant role in the trading and shipping of a wide range of goods, including various raw materials and semi-finished products.^38^ Numerous products, including cloth, salt, wax, copper, and iron, were exported between Scandinavian countries and the Baltic Sea ports.^47^ The port in Lübeck was one of the main markets for trading metals coming from Scandinavia and later, Spain. This was particularly the case for iron during the late Middle Ages.^48^ One of the main areas where iron ore was exploited at that time was the Bergslagen region, in south-central Sweden, constituting the largest concentration of base metal and iron ores in northern Europe.^49,50^ The iron extracted from this region is referred to as Osmund iron. It is documented that Osmund iron was exported in the form of bars, transported by sea in barrels, and then distributed further to smithies across Western Europe.^37^ Unfortunately, to the best of our knowledge, there are no isotopic data available for Osmund iron. There exists, however, information on the isotopic composition of several ore deposits in the Bergslagen region. Within this region, isotopic data for the Långban locality, an area rich in various types of ores, but primarily rich in iron and manganese oxides, reveal a ^206^Pb/^204^Pb ratio of 15.712 ± 0.012, ^207^Pb/^204^Pb ratio of 15.331 ± 0.015 and ^208^Pb/^204^Pb ratio of 32.191 ± 0.045. These ratios differ significantly from those obtained for the samples excavated at Hoeke. Although the data collected in the present study differ from that obtained for the Långban locality, we cannot definitively rule out the possibility that the iron originated from the Bergslagen region. Different regions with different geological units tend to have distinct isotopic signatures. It is known that even within a small geographical area, isotopic data can vary significantly due to the underlying geological processes. This variability makes it challenging to precisely pinpoint the provenance of the material examined.

Potential contamination during sampling, sample preparation and isotope ratio measurements was ruled out as the cause of the observed variability in the samples. All labware was thoroughly cleaned, and sample manipulation was performed in an evaporation box, which was demonstrated to provide low blank levels. Additionally, sample pretreatment was conducted with great care to avoid mixing different types of samples, thereby minimizing the risk of cross-contamination. Moreover, for these target elements, ball milling does not introduce any measurable contamination. The use of agate grinding heads, which are commonly employed in sample powder preparation, ensures that the samples are homogenized without detectable contamination.^51–53^

The large variation within our data could, therefore, potentially be attributed to the presence of Pb from different sources. Ores may have been extracted from distinct locations and subsequently blended during the iron production process. It is plausible to suggest that the isotopic signature observed in the iron slags from the archaeological site of Hoeke does not represent the isotopic signature of a single deposit, but rather a combination of metals sourced from different iron deposits. This large variation in isotopic data is also visible in Fig. 5, showing variability even within a single sample (Fig. 5).

In addition, it is possible that the iron ore used in Hoeke was a combination of material from different sources, in addition to the Bergslagen region. It is noteworthy that during the transit of iron to Belgium, there could have been potential intermediary points *en route* where mixing or transhipment of materials occurred. Although speculative, such scenarios could have contributed even more to the heterogeneity observed in this sample set.

In addition, the large variations in Pb isotope ratios could also be due to changes in the conditions during production. Historical iron production made use of open-air furnaces where emission rates of certain pollutants, such as Pb, and water quality were uncontrolled. As a result, “cross-contamination” between samples cannot be excluded. It is noteworthy that slags are the waste products of metal production and contain a range of impurities from every step of the operational chain. For example, the use of additives like flux can change the final composition of slags. Additionally, some slags might have been remelted by the smiths due to their high metal content, and the addition of other materials used during this process may alter the overall isotopic composition of the slag. These limitations have also been previously reported by various other authors, highlighting significant variation of Pb isotope ratios within a single sample set. Some studies have documented differences in Pb isotopic composition among various ore samples from within the same deposit.^54,55^ This variability makes the use of the Pb isotope ratios as a tool for provenancing iron artefacts challenging. For example, Hauptmann *et al.* emphasized the considerable variability in Pb isotopic composition in certain copper deposits located at Feinan (Jordan), making it difficult to establish a unique fingerprint for a specific location.^56^ However, in their study, combining this method with trace element data has proven effective in distinguishing between various mining districts.

Similar investigations have been conducted to determine whether lead from the same single ore deposit exhibits the same isotopic composition.^4^ Depending on the mining site, it can be observed that some show isotopic homogeneity, while others exhibit a significant variation in Pb isotope ratios. This variation is typically attributed to the fact that a large deposit may be the result of multiple mineralization processes and stages, leading to isotopic heterogeneities.^57^

Interpreting the Fe isotope ratio results poses an even greater challenge, primarily due to the limited amount of data in literature about the Fe isotopic composition of iron ores as a potential proxy for provenance in archaeology. There have been only a few studies so far dedicated to Fe isotopic analysis as a tool for provenancing iron specimens. Milot *et al.* examined ore, slags and metal samples from the Montagne Noire massif (SW of France) and obtained close-range results, suggesting that the Fe isotopic composition of ore is preserved throughout the iron production process, including smelting and smithing.^31,45^ However, there is a lack of data to ascertain whether the Fe isotopic composition undergoes significant changes during the preliminary treatment of iron ore (such as roasting).

The values obtained in this work for *δ*^56^Fe are spread over 0.4‰. This range is considerably larger than those observed for ores from other locations, such as the Montagne Noir or the Schwarzwald region.^32,45^ The iron found at Hoeke can thus represent a wide variety of mineralisation types or provenances. As a result, iron provenancing depending on iron isotope ratio data is not feasible in this case. However, it can assist in narrowing down the number of potential origins for the Fe ore.

The distinctive Fe isotopic variability observed within the collection of materials examined could additionally or alternatively also be attributed to redox processes occurring during mineralisation. For instance, in the case of bog iron ores, the isotopic signal is likely altered during the dissolution of the iron, which led to the intra-deposit variations.^32^ It is to note that within the scope of this study, it was not possible to determine whether fractionation occurred at the early stages of iron production process, given the unavailability of an ore sample for this sample set.

The provenance of coal has been previously established both by biostratigraphic analysis and by studying historical written sources, pointing to the Durham-Newcastle coalfield as a possible origin.^46^ The variation in ^206^Pb/^207^Pb isotope ratios for coal in this study is relatively small with a variation between 1.17 and 1.18 (*n* = 5) only. Comparing these data with the published Pb isotope ratios for coal in selected places in Europe (Table 5), confirms that the Hoeke coal could come from England. However, there is very little variation between coal from various locations in Europe, and ranges for coal from different locations mostly overlap. Despite the relatively narrow range in the Pb isotopic compositions experimentally obtained, identification of the material's source without an adequate context, based on isotopic study only, seemed impossible.

**Table:** The range of ^206^Pb/^207^Pb ratios from different locations in Europe in coal samples

Country of coal origin	^206^Pb/^207^Pb	Source
Spain	1.13–1.27	58
Scotland	1.16–1.19	54
Czech Republic	1.17–1.24	59
England and Wales	1.17–1.20	60
Ireland	1.17–1.31	60
Belgium	1.17–1.18	61
Switzerland	1.18	62
Poland	1.17–1.18	60
Portugal	1.18–1.20	63

The situation is different for the clay samples in this study, as their origin is expected to be local or from a not so distant location (within Flanders). During the iron production process, craftsmen commonly used local clay for constructing heating structures, such as furnaces and hearths.^64^ According to reference data,^65^ the coastal area of Belgium is characterized by the presence of Holocene sediments, with a ^87^Sr/^86^Sr ratio of 0.7092 (which is equal to that of contemporary ocean water). Nevertheless, the ^87^Sr/^86^Sr isotope ratio for clay excavated at Hoeke falls within the range of 0.713–0.718 which does not overlap with the coastal signal. Moreover, the Sr isotopic composition of clay overlaps with the range found for iron slags (Fig. 4). This isotopic heterogeneity in this sample set could thus be the result of mixing of Sr from various sources or potentially the (bidirectional) migration of Sr between the clay and the slag material.

Similarly, it was initially expected that hammer scales would exhibit a similar isotopic composition as the iron slags since they both originate from the same source – iron. However, this study reveals a significant spread in the isotopic composition of the elements studied for all materials examined. This suggests that during the production of certain objects, fragments of metal from different sources could have been remelted and combined to create a single new item. This process could potentially also explain the isotopic differences between the slag and hammer scales. Moreover, during the iron production process, the incorporation of materials like clay and coal might have introduced isotopic variability, resulting in the heterogeneity observed in the sample set, thereby explaining the observed overlap.^66^

The large spread in isotope ratios, which can be the result of the use of raw materials from different provenances and/or mixing of elements from various raw materials (ore, coal and clay) prevents solid conclusions to be drawn. Further investigation, involving the spatial distribution of isotope ratios within the samples, could reduce these limitations and provide a deeper understanding of the processes involved. In any case, it is clear that a combination of geochemical data with studies on the historical context is crucial for reconstructing the material's origin and drawing reliable conclusions.

## 5. Conclusions

This study explored methodological aspects for the characterization of Pb, Sr and Fe isotopic signatures of medieval iron slags for provenancing purposes. QA/QC indicated that accurate and precise results were obtained for these complex and heterogenous sample matrices despite the target elements being present in a wide range of concentrations. An additional interesting outcome of this study is that it has been shown that reliable results can be obtained when carrying out the sample preparation in an evaporation box used in a common lab.

Unfortunately, however, the Pb, Sr and Fe isotopic compositions of iron slags, hammer scales, clay, and coal exhibit variability, yet they cluster within a similar range. This observation suggests that the mixing of different materials during the iron production process could generate a relatively uniform range of isotopic compositions for the different types of materials within the sample set. Furthermore, it cannot be excluded that the use of different ore sources to produce iron might contribute to the isotopic variability as well. Additionally, the observed spread could also have been influenced by natural isotopic variations within ore deposits. The study's findings deepen our insight of medieval iron production and trade networks. The observed isotopic variability suggests expanding specialization, with each workshop focusing on a specific task, such as welding or bloom refining. Moving semi-finished products between these specialized locations could contribute to overall isotopic heterogeneity as the materials picked up impurities from each place. Furthermore, different ores could have been used to obtain the desired properties of the final product, thus demonstrating the progress of metal processing techniques used by medieval craftsmen. Acknowledging these aspects is crucial for interpreting isotope ratio results for the purpose of provenance analysis.

The determination of the provenance of iron from the late medieval port system of Hoeke is still uncertain, mainly due to the lack of primary ore samples. The access to and characterization of the primary ore samples is demonstrated to be of crucial importance to draw meaningful conclusions in this context. For this purpose, ore samples can be retrieved from sites identified by historical sources as potential locations or accessed from museums, which entails the need for destructive sampling of the specimens. Therefore, an interdisciplinary approach is necessary to address the challenges of metal provenance studies. As an additional consideration, establishing a database of isotopic compositions of iron ores from different regions would be valuable to determine the possible provenance of iron.

## Data availability

The authors believe that all relevant data have been made available in the manuscript and the corresponding ESI.† Should data be missing, these will be made available by the authors upon simple request.

## Author contributions

Conceptualization, P. B., W. D. C. and F. V.; methodology, P. B. and M. C.-R.; formal analysis, P. B. and M. C.-R.; resources, W. D. C. and F. V.; data curation, P. B and M. C.-R; writing—original draft preparation, P. B.; writing—review and editing, P. B., M. C.-R., J. D. G., S. D. and F. V.; visualization, P. B.; supervision, M. C.-R. and F. V.; project administration, W. D. C. and F. V.; funding acquisition, W. D. C. and F. V. All authors have read and agreed to the published version of the manuscript.

## Conflicts of interest

There are no conflicts to declare.

## Supplementary Material

RA-014-D4RA02887B-s001
