# Supplementary material for: Capabilities and limitations of Pb, Sr and Fe isotopic analysis of iron-rich slags: a case study on the medieval port at Hoeke (Belgium)
Source: RSC Adv. 2024 Jul 10;14(30):21887–900. doi: 10.1039/d4ra02887b (PMC11234174; doi:10.1039/d4ra02887b)
Supplement: RA-014-D4RA02887B-s001 [file RA-014-D4RA02887B-s001.pdf]

Electronic Supplementary Material (ESI) for RSC Advances.
This journal is © The Royal Society of Chemistry 2024

Capabilities and limitations of Pb, Sr and Fe isotopic analysis of iron-rich slags: a case study on the Medieval port at Hoeke (Belgium)

Paulina Biernacka,[a,b] Marta Costas-Rodríguez,[a,c] Wim De Clercq,[b] Stijn Dewaele[d], Johan De Grave[d], Frank Vanhaecke*[a]

[a] Ghent University, Department of Chemistry, Atomic & Mass Spectrometry – A&MS research unit, Campus Sterre, Krijgslaan 281 – S12, 9000 Ghent, Belgium.

[b] Historical Archaeology Research Group, Department of Archaeology, Ghent University, St.-Pietersnieuwstraat 35, 9000 Ghent, Belgium.

[c] Centro de Investigación Mariña, Universidade de Vigo, Departamento de Química Analítica y Alimentaria, Grupo QA2, 36310 Vigo, Spain.

[d] Laboratory for Mineralogy and Petrology, Department of Geology, Ghent University, Krijgslaan 281 S8, 9000, Ghent, Belgium

* Corresponding author's e-mail: frank.vanhaecke@ugent.be

Table S1: Lead and strontium concentrations and isotopic signatures of the iron slag, hammer scale, clay and coal samples investigated.

Sample	Type	Pb, µg g^{-1}	^{206}Pb/^{204}Pb	^{207}Pb/^{204}Pb	^{208}Pb/^{204}Pb	^{207}Pb/^{206}Pb	^{208}Pb/^{206}Pb	^{208}Pb/^{207}Pb	^{206}Pb/^{207}Pb	Sr, µg g^{-1}	^{87}Sr/^{86}Sr
1	slag	18.40	18.5107	15.6511	38.5591	0.8455	2.0831	2.4637	1.1827	45.56	0.7127
2	slag	73.12	18.3826	15.6118	38.3743	0.8493	2.0875	2.4580	1.1775	347.58	0.7100
3	slag	30.94	18.5831	15.6533	38.5862	0.8423	2.0764	2.4651	1.1872	94.39	0.7110
4	slag	11.77	18.6340	15.6703	38.5752	0.8409	2.0701	2.4617	1.1891	72.78	0.7220
5	slag	25.92	18.5129	15.6553	38.5776	0.8456	2.0838	2.4642	1.1825	82.30	0.7154
6	slag	67.20	18.6328	15.6677	38.7262	0.8409	2.0784	2.4717	1.1892	353.51	0.7102
7	slag	12.84	18.3973	15.6198	38.4460	0.8490	2.0898	2.4614	1.1778	82.44	0.7138
8	slag	198.02	18.4424	15.6303	38.4892	0.8475	2.0870	2.4625	1.1799	149.93	0.7189
9	slag	29.88	18.7021	15.6647	38.8211	0.8376	2.0758	2.4783	1.1939	160.01	0.7162
10	slag	68.68	18.8226	15.6765	38.8811	0.8329	2.0657	2.4802	1.2007	97.37	0.7175
11	slag	26.13	18.5356	15.6498	38.5258	0.8443	2.0785	2.4617	1.1844	179.40	0.7176
12	slag	12.65	18.5331	15.6668	38.5566	0.8453	2.0804	2.4610	1.1830	89.26	0.7108
041	slag	21.26	18.4369	15.6685	38.2733	0.8498	2.0759	2.4427	1.1767	158.13	0.7199
043	slag	19.26	18.4422	15.6681	38.4789	0.8496	2.0865	2.4559	1.1771	118.64	0.7172
047	slag	3.66	18.2230	15.6323	38.2629	0.8578	2.0997	2.4477	1.1657	48.55	0.7114
051	slag	31.33	17.9462	15.6327	37.9271	0.8711	2.1134	2.4261	1.1480	73.58	0.7148
041-A	slag	1.68	18.3346	15.6672	38.3650	0.8545	2.0925	2.4487	1.1703	77.98	0.7194
041-B	slag	2.95	18.4583	15.6691	38.4612	0.8489	2.0837	2.4546	1.1780	112.92	0.7195

Sample	Type	Pb, μg g^{-1}	$^{206}Pb/^{204}Pb$	$^{207}Pb/^{204}Pb$	$^{208}Pb/^{204}Pb$	$^{207}Pb/^{206}Pb$	$^{208}Pb/^{206}Pb$	$^{208}Pb/^{207}Pb$	$^{206}Pb/^{207}Pb$	Sr, μg g^{-1}	$^{87}Sr/^{86}Sr$
041-C	slag	0.88	18.4333	15.6727	38.4006	0.8502	2.0832	2.4502	1.1761	70.44	0.7196
042	slag	0.79	18.5558	15.6628	38.5285	0.8441	2.0764	2.4599	1.1847	26.15	0.7194
043-A	slag	4.41	18.3164	15.6508	38.3629	0.8545	2.0945	2.4512	1.1703	89.59	0.7166
043-B	slag	0.09	18.4256	15.6583	38.4545	0.8498	2.0870	2.4559	1.1767	14.25	0.7174
047	slag	3.20	18.2148	15.6294	38.3064	0.8581	2.1030	2.4509	1.1654	40.61	0.7106
048	slag	2.34	18.0888	15.6467	38.1303	0.8650	2.1080	2.4370	1.1561	143.67	0.7180
050	slag	4.40	18.4902	15.6680	38.5406	0.8474	2.0844	2.4598	1.1801	139.11	0.7154
051	slag	3.11	18.4072	15.6553	38.4472	0.8505	2.0887	2.4559	1.1758	86.25	0.7160
1.1.C.1	slag	65.79	18.6403	15.6609	38.6147	0.8402	2.0716	2.4657	1.1902	82.64	0.7111
1.1.C.1.A	slag	18.85	18.4364	15.6366	38.4086	0.8481	2.0833	2.4563	1.1791	23.92	0.7119
1.2.B	slag	26.68	18.5263	15.6554	38.5523	0.8450	2.0809	2.4626	1.1834	57.18	0.7153
1.2.C.1	slag	6.75	18.4699	15.6774	38.4605	0.8488	2.0823	2.4532	1.1781	8.30	0.7175
1.2.D.1	slag	15.84	18.4375	15.6787	38.4570	0.8504	2.0858	2.4528	1.1760	174.59	0.7211
1.2.D.2	slag	16.11	18.5552	15.6682	38.5707	0.8444	2.0787	2.4617	1.1843	98.62	0.7180
1.2.E.1	slag	11.82	18.3481	15.6314	38.3068	0.8519	2.0878	2.4506	1.1738	58.33	0.7154
1.2.G.2	slag	149.62	18.6900	15.6603	38.7431	0.8379	2.0729	2.4740	1.1935	21.94	0.7196
1.2.G.2.A	slag	118.23	18.5404	15.6699	38.6009	0.8452	2.0820	2.4634	1.1832	536.65	0.7194
2.B	slag	11.43	18.4544	15.6509	38.3893	0.8481	2.0802	2.4529	1.1791	96.98	0.7219
2.C.2	slag	7.36	18.5087	15.6282	38.3096	0.8444	2.0698	2.4513	1.1843	110.45	0.7193
2.D.1	slag	23.19	18.6623	15.6726	38.5702	0.8398	2.0667	2.4610	1.1908	95.46	0.7206
2.D.1.A	slag	12.28	18.5129	15.6601	38.4114	0.8459	2.0748	2.4528	1.1822	107.08	0.7206
2.E.1	slag	16.98	18.6480	15.6776	38.6948	0.8407	2.0750	2.4682	1.1895	120.58	0.7216
2.F.1.A	slag	51.01	18.5092	15.6528	38.5337	0.8457	2.0819	2.4618	1.1825	111.37	0.7155
2.G.3	slag	17.28	18.4821	15.6474	38.3270	0.8466	2.0737	2.4494	1.1812	112.44	0.7219
2.H.1	slag	5.45	18.3228	15.6735	38.2881	0.8554	2.0896	2.4429	1.1690	45.81	0.7190
2.H.1.A	slag	12.63	18.4239	15.6513	38.4063	0.8495	2.0846	2.4539	1.1771	45.93	0.7196
2.I	slag	12.62	18.4347	15.6447	38.3942	0.8486	2.0827	2.4541	1.1783	39.22	0.7154
2A	slag	14.69	18.5383	15.6727	38.5022	0.8454	2.0769	2.4566	1.1828	147.58	0.7180
2F	slag	25.01	18.5085	15.6680	38.5587	0.8465	2.0833	2.4610	1.1813	170.39	0.7154
PCB	slag	13.00	18.3947	15.6367	38.3420	0.8501	2.0844	2.4521	1.1764	95.52	0.7148
048-yellow	surface	5.04	18.5824	15.6668	38.6312	0.8431	2.0789	2.4658	1.1861	93.02	0.7138
051-yellow	surface	6.80	18.6530	15.6806	38.7283	0.8406	2.0763	2.4698	1.1896	305.87	0.7105
1.1.A	surface	25.46	18.4905	15.6620	38.6395	0.8470	2.0897	2.4671	1.1806	161.19	0.7110
1.1.A	surface	110.28	18.9191	15.6946	38.9533	0.8296	2.0589	2.4820	1.2054	56.43	0.7150

Sample	Type	Pb, µg g^{-1}	$^{206}Pb/^{204}Pb$	$^{207}Pb/^{204}Pb$	$^{208}Pb/^{204}Pb$	$^{207}Pb/^{206}Pb$	$^{208}Pb/^{206}Pb$	$^{208}Pb/^{207}Pb$	$^{206}Pb/^{207}Pb$	Sr, µg g^{-1}	$^{87}Sr/^{86}Sr$
1.1.A.1	surface	24.38	18.3985	15.6297	38.3480	0.8495	2.0843	2.4535	1.1771	136.05	0.7110
1.2.C.2	surface	6.65	18.5625	15.6739	38.4898	0.8444	2.0735	2.4557	1.1843	26.03	0.7164
1.2.E.1	surface	8.46	18.3481	15.6314	38.3068	0.8519	2.0878	2.4506	1.1738	602.93	0.7153
1.2.F	surface	109.87	18.5379	15.6208	38.4773	0.8426	2.0756	2.4632	1.1867	164.55	0.7173
1.2.F	surface	172.05	18.4921	15.6300	38.5036	0.8452	2.0822	2.4634	1.1831	1367.93	0.7164
1.2.F.1	surface	58.88	18.7570	15.6783	38.8025	0.8359	2.0687	2.4749	1.1964	51.14	0.7166
1.2.G.1	surface	23.19	18.3481	15.6314	38.3068	0.8519	2.0878	2.4506	1.1738	19.50	0.7136
2.B.2	surface	29.05	18.5157	15.6629	38.5510	0.8459	2.0821	2.4613	1.1821	42.81	0.7155
2.C.1	surface	15.09	18.5553	15.6684	38.5320	0.8444	2.0766	2.4592	1.1843	107.54	0.7190
2.G.1	surface	37.79	18.6171	15.6763	38.6409	0.8420	2.0756	2.4649	1.1876	101.11	0.7138
2.G.2	surface	15.83	18.4727	15.6881	38.5057	0.8493	2.0845	2.4544	1.1775	112.82	0.7220
2.G.2.A	surface	24.45	18.6424	15.6785	38.6107	0.8410	2.0711	2.4626	1.1890	89.49	0.7182
2.H.2	surface	17.76	18.6861	15.6799	38.5993	0.8391	2.0657	2.4617	1.1917	17.69	0.7180
2.I.2	surface	32.72	18.5664	15.6679	38.5202	0.8439	2.0747	2.4585	1.1850	252.77	0.7140
2.I.2	surface	35.09	18.6662	15.6697	38.6575	0.8395	2.0710	2.4670	1.1912	202.64	0.7173
1.2.G.1	surface	19.51	18.2674	15.6131	38.2545	0.8547	2.0941	2.4502	1.1700	350.57	0.7135
HH01	hammer scales	11.71	18.5719	15.6728	38.6313	0.8439	2.0801	2.4649	1.1850	52.77	0.7147
HH02	hammer scales	71.25	18.5681	15.6773	38.6273	0.8443	2.0803	2.4639	1.1844	30.39	0.7147
HH03	hammer scales	113.80	18.4712	15.6731	38.5323	0.8485	2.0861	2.4585	1.1785	650.99	0.7147
HH04	hammer scales	112.45	18.4188	15.6534	38.4446	0.8499	2.0872	2.4560	1.1767	622.46	0.7147
HH05	hammer scales	102.81	18.4670	15.6693	38.5134	0.8485	2.0855	2.4579	1.1785	222864.45	0.7106
1.2.B	clay	80.23	18.4101	15.6346	38.3759	0.8492	2.0845	2.4546	1.1775	122.63	0.7155
stone	clay	120.15	18.3814	15.6339	38.4528	0.8505	2.0919	2.4596	1.1757	169366.87	0.7135
1.2.B	clay	56.03	18.7090	15.6649	38.8867	0.8373	2.0785	2.4824	1.1943	125.87	0.7164
1.2.F	clay	191.90	18.6785	15.6502	38.7055	0.8379	2.0722	2.4732	1.1935	237.52	0.7174
FL	clay	22.92	18.6723	15.6848	38.8381	0.8400	2.0800	2.4762	1.1905	120.69	0.7178
FL.1	clay	137.48	18.4363	15.6197	38.3955	0.8472	2.0826	2.4581	1.1803	185.63	0.7137
stone	clay	80.89	18.4480	15.6703	38.5501	0.8494	2.0897	2.4601	1.1773	124.58	0.7162
coal01	coal	79.07	18.4552	15.6687	38.5880	0.8490	2.0909	2.4627	1.1778	75.81	0.7162
coal02	coal	78.69	18.4583	15.6726	38.5971	0.8491	2.0910	2.4627	1.1777	1326.55	0.7123
coal03	coal	91.41	18.3171	15.6612	38.4360	0.8550	2.0983	2.4542	1.1696	923.82	0.7161
coal04	coal	116.04	18.3832	15.6612	38.5021	0.8519	2.0944	2.4584	1.1738	919.63	0.7123
coal05	coal	60.38	17.9380	15.6496	38.2337	0.8722	2.1308	2.4431	1.1462	609.69	0.7147

Sample	Type	Pb, µg g^{-1}	$^{206}Pb/^{204}Pb$	$^{207}Pb/^{204}Pb$	$^{208}Pb/^{204}Pb$	$^{207}Pb/^{206}Pb$	$^{208}Pb/^{206}Pb$	$^{208}Pb/^{207}Pb$	$^{206}Pb/^{207}Pb$	Sr, µg g^{-1}	$^{87}Sr/^{86}Sr$
a = 1SD<br>b = 2SD		7.03 %	0.0345[a]	0.0275[a]	0.0675[a]	0.0005[a]	0.0007[a]	0.0007[a]	0.0001[a]	9.04 %	0.0001[b]

Table S2: Fe concentration and isotopic signature of the iron slag, hammer scale, clay and coal samples investigated.

Sample	Type	Fe, wt %	δ^{56} Fe, ‰	δ^{57} Fe, ‰
041	slag	32.2	0.065	0.117
043	slag	55.1	-0.174	-0.189
047	slag	49.2	-0.083	-0.154
051	slag	71.8	-0.142	-0.172
041-A	slag	26.1	0.232	0.306
041-B	slag	27.4	0.037	0.068
041-C	slag	24.3	0.097	0.132
042	slag	50.4	-0.288	-0.487
043-A	slag	44.6	-0.147	-0.224
043-B	slag	23.4	-0.121	-0.158
047	slag	50.3	-0.054	0.065
048	slag	44.3	-0.073	-0.121
1.2.D.1	slag	22.2	-0.025	-0.079
2.H.1	slag	57.5	-0.228	-0.307
2F	slag	42.2	0.111	0.164
048	surface	46.3	-0.175	-0.187
1.1.A	surface	43.6	-0.138	-0.227
2.G.2	surface	28.7	-0.219	-0.230
2.I.2	surface	11.6	-0.165	-0.207
HH	hammer scales	37.7	-0.228	-0.298
HH	hammer scales	46.0	-0.287	-0.423
FL	clay	8.2	0.005	0.052
stone	clay	10.5	-0.236	-0.278
coal	coal	0.9	-0.001	0.028
±2SE		4.5 %	0.042	0.063
